# Effects of kangaroo mother care restrictions during the COVID-19 pandemic on feeding and physiological parameters in preterm neonates: a cohort study

**DOI:** 10.1590/1806-9282.20240864

**Published:** 2025-03-17

**Authors:** Zühal Çamur, Deniz Akyildiz

**Affiliations:** 1 Karabük University, Faculty of Health Sciences, Department of Midwifery – Karabük, Turkey.; 2 Kahramanmaraş Sütçü İmam University, Faculty of Health Sciences, Department of Midwifery – Kahramanmaraş, Turkey.

**Keywords:** Kangaroo mother care, Newborn, Neonatal intensive care unit, Preterm, COVID-19

## Abstract

**OBJECTIVE:**

This research investigates the impact of kangaroo mother care restrictions on feeding and physiological parameters in preterm newborns during the COVID-19 pandemic.

**METHODS:**

A retrospective cohort design was used, including 169 preterm neonates born at 30–34 weeks of gestation, recruited from a Neonatal Intensive Care Unit in Denizli, Turkiye. The study compared a kangaroo mother care group (n=78) and a kangaroo mother care-restricted group (n=91). Data analysis was conducted using the chi-square test, Fisher's exact test, and t-test.

**RESULTS:**

The average duration until the initiation of full oral feeding was shorter in the kangaroo mother care group (mean difference=4.58, 95%CI 0.61–8.43, p=0.020). Newborns in the kangaroo mother care-restricted group had a higher likelihood of reverting to gavage feeding (OR 6.59, 95%CI 2.98–14.58, p=0.000), and higher withdrawal rates (OR 3.36, 95%CI 1.59–7.09, p=0.001). Newborns in the kangaroo mother care group experienced significantly lower rates of apnea attacks (OR 3.29, 95%CI 1.52–7.09, p=0.002), tachycardia (OR 4.43, 95%CI 1.54–12.76, p=0.004), and desaturation (OR 3.43, 95%CI 1.70–6.93, p=0.000).

**CONCLUSION:**

This study highlights the positive effects of kangaroo mother care on preterm newborns’ feeding and physiological parameters. It is recommended that kangaroo mother care be consistently provided to all newborns, even during exceptional circumstances such as pandemics.

## INTRODUCTION

Preterm births pose a significant health challenge globally, with adequate nutrition being a primary concern for these infants^1^. Kangaroo mother care (KMC) is an approach involving early and prolonged skin-to-skin contact with the mother or a substitute caregiver, along with exclusive and frequent breastfeeding^2^. Numerous studies have demonstrated the benefits of KMC, including improved temperature regulation, reduced infection rates, early discharge, enhanced neurodevelopment, and stabilized physiological parameters^3,4^.

In recent years, international health authorities and scientific communities have increasingly emphasized the importance of KMC in caring for preterm babies^1^. However, the COVID-19 pandemic has disrupted essential interventions like breastfeeding and KMC^5^. Evidence-based practices such as KMC were discontinued, leading to the separation of the mother–infant dyad in both COVID-19-positive mothers and those with unknown status^6^. Studies have shown significant reductions in the duration of KMC application during the pandemic, with some regions completely depriving preterm babies of KMC^7^. This increases the risk of mortality significantly, with a risk 65 times higher than that of death due to COVID-19 infection in newborns^8^. Furthermore, the same study reported that a 50% reduction in KMC application could lead to an additional 12,570 deaths in preterm infants, and a complete cessation could result in 25,140 additional deaths, along with a 2.3–4.6% rise in neonatal mortality across 127 countries^8^.

The pandemic has presented challenges for healthcare professionals and parents, with a lack of scientific evidence initially hindering effective response strategies^9^. Consequently, a spectrum of restrictive policy measures, ranging from general measures to quarantine protocols, became necessary to curb the spread of the virus. Restrictive policy measures profoundly impacted parental involvement in Neonatal Intensive Care Unit (NICU) settings, particularly affecting preterm infants requiring prolonged hospitalization^8^. Given the challenges posed by the pandemic, alternative methods such as allowing fully vaccinated parents to participate in KMC while using personal protective equipment or creating controlled KMC settings within NICUs could have been considered. Additionally, virtual support through telehealth for parents who were unable to be physically present may have helped maintain some aspects of KMC. These adaptations could have mitigated the impact of KMC restrictions while ensuring the safety of both infants and caregivers.

Under these stringent quarantine measures, preterm infants were deprived of KMC^10^. Although limited in number, studies investigating the impact of KMC restrictions on preterm infants during the pandemic period are emerging. For instance, a study comparing preterm infants in NICUs during the COVID-19 pandemic and preceding periods revealed a shorter duration until initiation of full oral feeding in the pre-pandemic period^7^.

Identifying the adverse effects of KMC restrictions on newborns during the pandemic is imperative for future preparedness plans and neonatal care practices. Therefore, this study explores the effects of KMC restrictions on feeding, physiological parameters, and treatment processes of preterm newborns during the COVID-19 pandemic.

## METHODS

A retrospective cohort study was conducted at a level III NICU in a state hospital in Denizli, Turkiye. Preterm infants born between 30 and 34 gestational weeks and admitted to the NICU were eligible. The study adhered to the STROBE guidelines^11^.

The minimum sample size was determined based on previous NICU discharge times^12^, resulting in a sample of 169 preterm newborns (78 in the KMC group and 91 in the KMC-restricted group). The KMC group included newborns hospitalized before the pandemic (March 10, 2019, to March 10, 2020), and the KMC-restricted group included those hospitalized during the pandemic (March 11, 2020, to March 11, 2021).

The first COVID-19 case in our country was detected on March 11, 2020. Subsequently, a pandemic was declared, leading to the discontinuation of KMC practice for newborns as a precautionary measure to mitigate the risk of transmission in the NICU where the study was conducted. Prior to the pandemic, mothers were accommodated in the hospital's maternal hotel and were regularly escorted to the unit every 3 h for breastfeeding and KMC sessions. Mothers typically visited the unit approximately 4–8 times daily, with KMC sessions lasting approximately 30–60 min each. However, due to the hospital's designation as a pandemic facility, maternal access to the NICU was restricted entirely during COVID-19. Consequently, breastfeeding and KMC practices were suspended for newborns during this time.

The study's KMC group (n=78) comprised newborns hospitalized in the NICU who had undergone KMC 1 year preceding the COVID-19 pandemic, from March 10, 2019, to March 10, 2020. Conversely, the KMC-restricted group (n=91) consisted of preterm newborns hospitalized in the NICU who did not receive KMC during the COVID-19 pandemic from March 11, 2020, to March 11, 2021.

Newborns born between 30 and 34 weeks of gestation, without facial deformities, and lacking respiratory, cardiovascular, gastrointestinal, and neurological disorders, were included. Excluded were newborns referred to another facility before follow-up completion, those whose mothers contracted COVID-19 during pregnancy or had active infections, and those developing pathologies hindering oral feeding.

Data were collected between October and December 2021 using a "Data Collection Form," with 24 questions about the newborns’ characteristics, nutrition, physical health, and discharge process. This form consists of 24 questions developed by researchers based on the literature^7,10^. The form included questions about the introductory characteristics of the newborns, nutrition, physical health, and the discharge process. The research data were collected by the first author. During the data collection process, the first author was employed as a nurse in the NICU where the study was conducted.

### Statistical analysis

Descriptive statistics, chi-square test, Fisher's test, and t-tests were used. Results were expressed as odds ratios (ORs) with 95% confidence intervals (CIs). Analyses were performed using SPSS 22 (IBM), with p-values <0.05 considered significant.

## RESULTS

Baseline characteristics such as maternal age, gestational age, birth weight, Apgar scores, gender, diagnosis of hospitalization, and therapy received did not differ significantly between the KMC and KMC-restricted groups (p>0.05). The groups were similar in terms of mechanical ventilation time, discharge time, and body weight at discharge from the NICU (p>0.05) (Table 1).

**Table 1:** Comparison of characteristics of newborns in kangaroo mother care and kangaroo mother care-restricted groups.

	KMC group (n=78)	KMC-restricted group (n=91)	MD [95%CI]	p-value*
**Continuous variables (mean**±**SD)**				
Maternal age	28.03±5.84	28.00±6.86	-0.03 [-1.96 to 1.89]	0.108
Birth weight (g)	1,835.71±327.42	1,880.60±421.83	44.88 [-69.02 to 158.80]	0.080
Gestation week	32.57±1.39	32.55±1.37	-0.02 [-0.44 to 0.40]	0.864
First-minute Apgar score	7.63±1.29	7.62±1.57	-0.01 [-0.44 to 0.42]	0.074
Fifth-minute Apgar score	9.02±0.94	9.07±0.99	0.05 [-0.22 to 0.33]	0.444
MV duration (days)	2.11±2.52	1.58±1.59	0.53 [-0.05 to 1.19]	0.099
Discharge time (days)	26.63±13.31	26.43±13.10	-0.20 [-4.27 to 3.78]	0.921
Body weight at discharge (g)	2,387.94±246.70	2,315.21±317.68	72.72 [-15.24 to 157.06]	0.103
**Categorical variables (%)**			**OR [95%CI]**	**p-value** **
Female	45 (49.5)	29 (37.2)	0.60 [0.32–1.12]	0.109
Vaginal birth	27 (29.7)	16 (20.5)	0.61 [0.30–1.24]	0.173
MV needs	65 (71.4)	63 (80.8)	1.68 [0.81–3.46]	0.158
Invasive MV	11 (16.9)	8 (12.7)	0.71 [0.26–1.91]	0.452
Need for surfactant	23 (25.3)	20 (25.6)	1.01 [0.50–2.04]	0.957

MD: mean difference; CI: confidence interval; OR: odds ratio; SD: standard deviation; MV: mechanical ventilation; KMC: kangaroo mother care.

* t-test.

** Chi-square test.

No difference was found in the time to start oral feeding between the groups. However, a significant difference was observed in the time to start full oral feeding, with the KMC group achieving this sooner (mean difference [MD] 4.58, 95%CI 0.61–8.43, p<0.05). The KMC-restricted group had higher rates of returning to gavage feeding (OR 6.59, 95%CI 2.98–14.58, p<0.001) and developing gastric residue (OR 3.36, 95%CI 1.59–7.09, p<0.05) (Table 2).

**Table 2:** Comparison of feeding characteristics of newborns in kangaroo mother care and kangaroo mother care-restricted groups.

	KMC group (n=78)	KMC-restricted group (n=91)	MD [95%CI]	p-value*
**Continuous variables (mean**±**SD)**				
Time to start oral feeding (days)	12.61±10.83	15.10±12.47	2.48 [-1.07 to 6.28]	0.167
Time to start full oral feeding (days)	17.05±11.97	21.64±13.34	4.58 [0.61 to 8.43]	0.020
**Categorical variables, n (%)**			**OR [95%CI]**	**p-value** **
Returned to gavage	10 (11.0)	35 (44.9)	6.59 [2.98–14.58]	0.000
Residue	13 (14.3)	28 (35.9)	3.36 [1.59–7.09]	0.001

KMC: kangaroo mother care; MD: mean difference; CI: confidence interval; OR: odds ratio; SD: standard deviation.

* t-test.

** Chi-square test. p<0.05 data are indicated in bold.

The groups were similar in terms of development of bradycardia. However, the KMC-restricted group had higher rates of apnea attacks (OR 3.29, 95%CI 1.52–7.09, p<0.05), tachycardia (OR 4.43, 95%CI 1.54–12.76, p<0.05), and desaturation episodes (OR 3.43, 95%CI 1.70–6.93, p<0.001) (Table 3).

**Table 3:** Comparison of physiological parameters of newborns in kangaroo mother care and kangaroo mother care-restricted groups.

	KMC group (n=78)	KMC-restricted group (n=91)	OR [95%CI]	p-value*
**Variables, n (%)**				
With episodes of bradycardia	2 (2.2)	5 (6.4)	3.04 [0.57–16.17]	0.251†
With episodes of apnea	12 (13.2)	26 (33.3)	3.29 [1.52–7.09]	**0.002**
With tachycardia episodes	5 (5.5)	16 (20.5)	4.43 [1.54–12.76]	**0.004** †
With episodes of desaturation	16 (17.6)	33 (42.3)	3.43 [1.70–6.93]	**0.000**

KMC: kangaroo mother care; OR: odds ratio; CI: confidence interval; SD: standard deviation.

* Chi-square test.

† Fisher's exact test.

* p<0.05 are indicated in bold.

## DISCUSSION

This study compared the outcomes of preterm newborns who underwent KMC restrictions during the COVID-19 pandemic with those who received KMC before the pandemic. The main focus was on feeding and physiological parameters. KMC has become standard care in nearly all NICUs, but restrictions during the pandemic were implemented to prevent transmission. These research findings are valuable for shedding light on how the KMC restriction initiative impacts preterm infants in NICUs during the pandemic.

Our study found that the time to achieve full oral feeding in preterm infants in the KMC group before the pandemic was approximately 4.5 days shorter than that in the KMC-restricted group. Additionally, higher rates of returning to gavage and developing gastric residue were noted in the KMC-restricted group. Similarly, a study conducted in India comparing preterm infants with restricted KMC during the COVID-19 pandemic to those in the pre-pandemic period found that the time to start feeds was significantly longer during the pandemic^7^. Various studies conducted with preterm infants have shown that the transition to full enteral feeding is shorter, and nutritional problems are significantly lower in the KMC group compared with the standard care group^13,14^. This can be attributed to the positioning provided by KMC, which reduces stress levels in newborns and enhances their digestion and nutrient metabolism^15^. One study reported that the KMC position reduced gastric residual volume, improving feeding tolerance^16^. Research indicates that KMC may serve as a method to reduce stress in newborns, significantly lowering cortisol levels^13,17^. However, a study comparing preterm infants in the NICU during the COVID-19 pandemic and before reported that newborns achieved full oral feeding in a shorter time in the pre-pandemic period^7^. These findings underscore the importance of KMC in improving nutritional outcomes for preterm infants and suggest its application should be sustained even during pandemics.

Furthermore, our research found that compared with the KMC-restricted group, newborns who underwent KMC before the pandemic experienced lower rates of apnea attacks, tachycardia, and desaturation episodes. Previous studies also demonstrate that newborns receiving KMC have lower rates of apnea and tachycardia during hospitalization compared with those without KMC^18,19^. This may be attributed to KMC inducing a decrease in average heart rate and promoting a calming and relaxing effect on newborns^19^. Maintaining the newborn in a calm and comfortable state leads to improved tissue oxygenation^20^, likely reducing oxygen consumption. Additionally, the upright position provided to the newborn during KMC optimizes respiratory function^21^. These findings highlight the positive effects of KMC on newborns’ physiological health indicators and advocate for its continuation during pandemic situations.

In a risk analysis study in the literature, it was revealed that in the worst-case scenario (100% transmission) for preterm newborns, COVID-19 could result in the death of an estimated 1,950 newborns. Conversely, when KMC is applied, the lives of 125,680 newborns could be saved. Therefore, the study concluded that the benefit of KMC was 65 times higher than the risk of death from COVID-19^8^. In the same study, it was reported that a 50% reduction in KMC application could lead to 12,570 additional deaths in preterm infants, and a complete cessation could result in 25,140 additional deaths and a 2.3–4.6% increase in neonatal mortality across 127 countries^8^. These findings underscore the critical importance of KMC for newborn health. It is imperative to make arrangements to ensure the continuation of KMC implementation, especially in planning newborn care during extraordinary situations such as pandemics.

Our research found that preterm newborns who underwent KMC before the pandemic and those who were KMC-restricted during the pandemic were similar in terms of body weight at discharge. In another study conducted during the COVID-19 pandemic, the discharge weight of preterm infants with restricted KMC was found to be similar to that of infants in the pre-pandemic period^7^. Similarly, another study observed no difference in weight gain between newborns who received KMC and those who were KMC-restricted^14^. Conversely, various studies have reported the effectiveness of KMC on weight gain in preterm infants^22,23^. This variation in findings may be attributed to other variables, such as intravenous fluids and treatments, influencing newborns’ weight gain in NICUs^22^.

Furthermore, KMC is known to increase breastfeeding rates^13^. However, since our study was conducted during the COVID-19 pandemic, restrictions on mothers’ entry to intensive care units and limitations on breastfeeding may have mitigated its effects on weight gain in newborns. Nonetheless, the role of parents in newborn care is crucial, and considering the available evidence and the risk of transmission, it is concluded that mother–infant separation cannot be justified and may even be harmful.

### Limitations

The first limitation of our study is that the results cannot be generalized since the research was conducted in a single center. Second, data on newborns’ health, growth, development, and nutritional outcomes were not collected prospectively after discharge.

Another limitation is the lack of control over certain variables during the COVID-19 pandemic, such as staff availability, NICU resources, and changes in care practices. These factors, influenced by the pandemic, may have impacted the outcomes and should be considered when interpreting the results. Future studies should aim to account for these variables more effectively.

## CONCLUSION

The main findings of this study indicate that preterm infants subjected to KMC restrictions during the pandemic experienced delays in achieving full oral feeding compared with those who received KMC before the pandemic. Additionally, they exhibited a higher incidence of reverting to gavage feeding and developing gastric residue. The study highlights the critical importance of KMC for newborn health and the need to prioritize its implementation even during extraordinary circumstances like pandemics.
